# (6,7-Dimethoxy-4-methylisoquinolinyl)-(4’-methoxyphenyl)-methanone, a New Benzylisoquinoline Alkaloid from *Beilschmiedia brevipes*

**DOI:** 10.3390/molecules15042339

**Published:** 2010-03-31

**Authors:** Pratiwi Pudjiastuti, Mat Ropi Mukhtar, A. Hamid A. Hadi, Nurdin Saidi, Hiroshi Morita, Marc Litaudon, Khalijah Awang

**Affiliations:** 1 Department of Chemistry, Faculty of Science and Technology, University Airlangga, Surabaya, Indonesia; E-Mail: tiwi2000@hotmail.com (P.P.); 2 Centre for Natural Products and Drug Discovery, Block D, Department of Chemistry, Faculty of Science, University of Malaya, 50603 Kuala Lumpur, Malaysia; E-Mails: khalijah@um.edu.my (K.A.); ahamid@um.edu.my (A.H.A.H.); 3 Department of Chemistry, Faculty of Science and Mathematics, University of Syah Kuala, Banda Aceh, Indonesia; E-Mail: noersaidi@yahoo.com (N.S.); 4 Faculty of Pharmaceutical Sciences, Hoshi University, Ebara 2-4-41 Shinagawa, Tokyo 142-8501, Japan; E-Mail: moritah@hoshi.ac.jp (M.H.); 5 Centre de Recherche de Gif, Institut de Chimie des Substances Naturelles, CNRS, 1, Avenue de la Terrasse, 91198 Gif-sur-Yvette Cedex, France; E-Mail: marc.litaudon@icsn.cnrs-gif.fr (M.L.)

**Keywords:** *Beilschmiedia brevipes*, benzylisoquinoline, lauraceae, NMR

## Abstract

The leaves of *Beilschmiedia brevipes* provided a new benzylisoquinoline alkaloid: (6,7-dimethoxy-4-methylisoquinolinyl)-(4’-methoxyphenyl)-methanone (**1**) and *O*,*O*-dimethylannocherin A (**2**), a new natural compound which has been synthesized before. Complete ^1^H- and ^13^C-NMR data of both compounds were reported. The structures were established through various spectroscopic methods notably 1D- and 2D-NMR, UV, IR and HRESIMS.

## 1. Introduction

The production of phytochemicals such as flavonoids, endiandric acid derivatives, essential oils, fatty acids, epoxyfuranoid lignans and alkaloids by the *Beilschmiedia* species; *B. miersii*, *B. anacardioides*, *B. alloiophylla*, *B. brenesii*, *B. costaricensis*, *B. tilaranensis*, *B. jacques felixii*, *B. erythrophloia*, *B. madang*, *B. oreophila schlechter*, *B. podagrica* and *B. elliptica* has been the subject of a number of comprehensive articles [1,2,3,4,5,6,7,8,9,10,11]. This genus is also known to produce a large number of biologically active compounds possessing interesting skeletons [2,3,4]. In fact, the endiandric acid derivatives and epoxyfuranoid lignans have shown strong antibacterial and antitubercular activities [3,4,6].

In a continuing search for bioactive compounds and interesting chemical entities from Malaysian flora, our group has performed a phytochemical study on the leaves of a Malaysian Lauraceae, *Beilschmiedia brevipes*, which has led to the isolation of one new benzylisoquinoline, (6,7-dimethoxy-4-methylisoquinolinyl)-(4’-methoxyphenyl)-methanone (**1**) and one new natural product; *O*,*O*-dimethylannocherin A (**2**). In addition to these two compounds five other known alkaloids: *O*,*O*-dimethylcoclaurine [12], (6,7-dimethoxyisoquinolinyl)-(4’-methoxyphenyl)-methanone (**3**) [13], *O*-methylvelucryptine [14], armepavine [15], and norarmepavine [16] were also isolated. These alkaloids were obtained from the CH_2_Cl_2_ extract of the leaves of *Beilschmiedia brevipes.* This communication reports the isolation and complete ^1^H and ^13^C assignments of **1** and **2**. 

**Figure 1:**
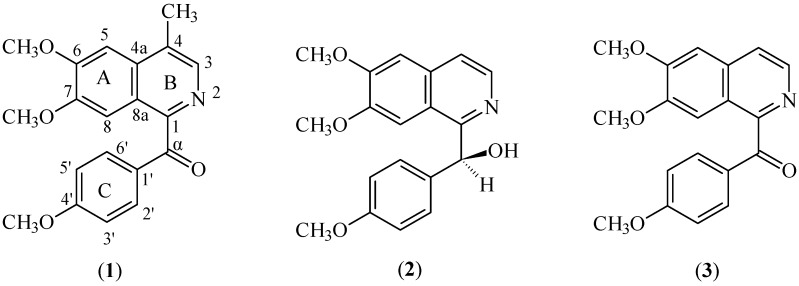
Structure of alkaloids **1–3**.

## 2. Results and Discussion

**Figure 2:**
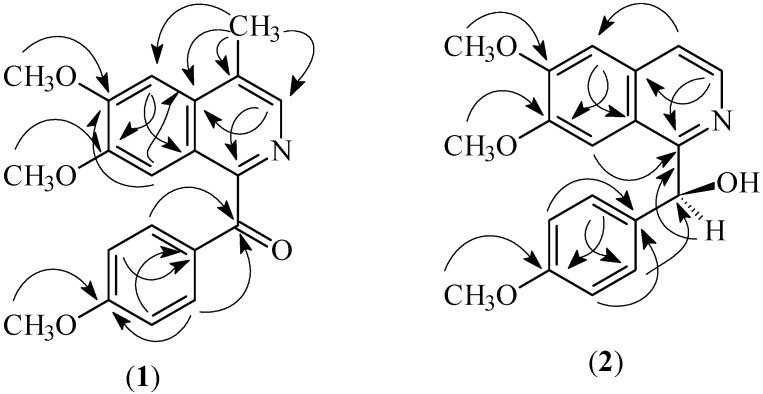
Selected HMBC correlation of alkaloids (**1**) and (**2**).

(6,7-Dimethoxy-4-methylisoquinolinyl)-(4’-methoxyphenyl)-methanone (**1**) was obtained as a brownish amorphous solid. The HRESIMS revealed a pseudomolecular ion peak at *m*/*z* 338.1373 [M + H]^+^, thus suggesting a molecular formula of C_20_H_20_NO_4 _(calc. 338.1392). The IR spectrum revealed absorption bands at 1,597 and 1,660 cm^−1^ due to the C=N and the C=O stretching vibrations respectively [17]. The ^1^H-NMR spectrum exhibited three singlets representing methoxyl groups at δ 4.12, 3.99 and 3.93 that could be assigned to C-6, C-7 and C-4’, respectively. A methyl singlet was observed at δ 2.71 and it correlated to C-4 (δ 128.2) and C-4a (132.9) respectively in the HMBC spectrum (Fig. 2), thus implying that the methyl is attached to C-4. The adjacent H-3 appeared as a singlet at δ 8.24. Two singlets were apparent at δ 7.24 and 7.58 corresponding to H-5 and H-8 respectively. This signal is commonly found in a 6,7-disubstituted α-oxobenzylisoquinoline [17,18]. In addition, two doublets of an AA’BB’ spin system appeared at δ 7.90 and 6.90 (*J =* 10.9 Hz) assignable to H-2’/6’ and H-3’/5’ respectively. 

^1^H- and ^13^C-NMR data (Table 1) indicated the presence of seven sp^2^ methines, eight sp^2^ quaternary carbons, one carbonyl and four methyl groups. The imine carbon (C-1) resonated at δ 152.7 and the adjacent carbonyl (C-α) appeared at δ 194.0. The quaternary carbon, C-4’, gave a signal at δ 163.8. The ^13^C-NMR analysis by Anita *et al.* [13] on papaveraldine and its related compounds showed that C-4’ bearing a methoxyl group would resonate at ~ δ 163.0, more deshielded than the other methoxylated sp^2^ carbons which usually resonate at ~ δ 149.0–153.0. Thorough analysis of the COSY, HMQC and HMBC spectra allowed the complete assignments of all protons and carbons of (6,7-dimethoxy-4-methylisoquinolinyl)-(4’-methoxyphenyl)-methanone (**1**) (Table 1).

The alkaloid *O*,*O*-dimethylannocherin A (**2**), 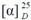 = +91° (*c* = 0.22, MeOH), was obtained as a brownish amorphous solid. The HRESI mass spectrum showed a molecular ion peak at m/z 326.1396 [M+H]^+^, thus giving a molecular formula of C_19_H_20_NO_4 _(calc. 326.1392 Δ 0.4 mmu). One fragmentation peak was observed at m/z 308.1457, [M-H_2_O]^+ ^which can be attributed to the loss of a water molecule thus suggesting the presence of an aliphatic hydroxyl group. The IR spectrum revealed absorption bands at 3387 and 1597 cm^−1^ for –OH and C=N stretching vibrations [17,18]. 

The ^1^H-NMR spectrum showed a singlet at δ 7.05 ascribable to H-3. A singlet representing C-8 proton appeared at δ 7.11 thus, suggesting that ring A was tetrasubstituted. A pair of doublets with a coupling constant of 5.6 Hz appeared at δ 8.38 and 7.52 which belong to H-3 and H-4 respectively [14]. In addition, an imine carbon signal (C-1) appeared at δ156.6. HMBC spectrum showed correlation between H-α at δ 6.23 and C-1 (δ 156.6) thus, indicating that ring B is fully unsaturated. Two doublets at δ 7.24 and 6.78 (*J* = 8.4 Hz, each two protons) are also observed for a disubstituted ring C. 

The ^13^C-NMR spectrum of (**2**) showed the presence of 19 carbons, which was in agreement with the molecular formula. The C-α hydroxyl peak was observed at δ 72.1. The complete ^1^H- and ^13^C-NMR spectral assignment of (**2**) was accomplished by thorough analysis of DEPT, COSY, HMQC, and HMBC data. The absolute configuration at C-α is determined as *S* due to the positive sign of the specific rotation for alkaloid (**2**) 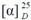 = +91° (*c* = 0.22, MeOH) [18,19].

**Table 1:** ^1^H-NMR (400 MHz) and ^13^C-NMR (100 MHz) spectral data of compounds **1**,**2 **and **3 **in CDCl_3_ (*δ* in ppm, *J* in Hz).

	Alkaloid					
Positions	(1)	(2)	(3)	(1)	(2)	(3)*
1				152.7	156.6	153.5
α		6.23 *s*		194.0	72.1	187.0
3	8.24 *s*	8.40 *d* (6.0)	8.42 *d* (5.6)	140.0	137.5	139.9
4		7.52 *d* (6.0)	7.53 *d* (5.6)	128.2	120.0	121.1
4a				132.9	133.9	133.8
5	7.24 *s*	7.06 *s*	6.61 *s*	101.4	105.3	104.7
6				152.1	153.1	153.0
7				150.4	150.1	150.9
8	7.58 *s*	7.11 *s*	7.20 *s*	104.7	103.5	104.0
8a				122.7	120.8	122.6
1′				129.9	135.2	129.7
2′	7.90 *d* (10.9)	7.24 *d* (8.4)	7.92 *d* (8.0)	133.1	128.9	133.1
3′	6.90 *d* (10.9)	6.78 *d* (8.4)	6.93 *d* (8.0)	113.5	114.1	113.6
4′				163.8	159.2	163.0
5′	6.90 *d* (10.9)	6.78 *d* (8.4)	6.93 *d* (8.0)	113.5	114.1	113.6
6′	7.90 *d* (10.9)	7.24 *d* (8.4)	7.92 *d* (8.0)	133.1	128.9	133.1
CH_3_-4	2.71 *s*			16.4		
OMe-6	4.12 *s*	3.97 *s*	4.03 *s*	55.9	56.1	56.0
OMe-7	3.99 *s*	3.78 *s*	3.93 *s*	55.4	55.9	56.0
OMe-4′	3.93 *s*	3.72 *s*	3.85 *s*	55.3	55.2	55.6

* ^13^C-NMR data are reproduced from Anita *et al*. [13].

### Biological Activity

The six alkaloids isolated in this study were tested for cytotoxic activity against P-388 murine leukemia cell lines. Only *O*,*O*-dimethylcoclaurine [12] exhibited any significant cytotoxicity, with an IC_50 _of 6.5 μg/mL (Table 2). 

**Table 2:** *In vitro* cytotoxic activity against P388 cell lines.

Compounds	IC_50_ (μg/mL)
(6,7-Dimethoxy-4-methylisoquinolinyl)-(4’-methoxyphenyl)-methanone (1)	>100
(6,7-Dimethoxyisoquinolinyl)-(4’-methoxyphenyl)-methanone (3) [13]	18.7
*O*,*O*-Dimethylcoclaurine [12]	6.5
*O*-Methylvelucryptine [14]	17.3
Armepavine [15]	42.2
Norarmepavine [16].	44.5

## 3. Experimental

### 3.1. General

The optical rotations were recorded on a Jasco (Japan) P1010 instrument equipped with a tungsten lamp. HRMS were obtained on a Thermo Finnigan Automass Multi. The ultraviolet spectra were obtained in MeOH on a Shimadzu UV-310 ultraviolet-visible spectrometer. The Fourier Transform Infrared (FTIR) spectra were obtained with CHCl_3_ (NaCl window technique) on a Perkin Elmer 2000 instrument. The ^1^H-NMR and ^13^C-NMR spectra were recorded in deuterated chloroform on a JEOL 400 MHz (unless stated otherwise) instrument; chemical shifts are reported in ppm on δ scale, and the coupling constants are given in Hz. 

Silica gel 60, 70–230 mesh ASTM (Merck 7734) was used for column chromatography. Mayer’s reagent was used for alkaloid screening. TLC aluminium sheets (20 × 20 cm Silica gel 60 F_254_) were used in the TLC analysis. The TLC spots were visualized under UV light (254 and 366 nm) followed by spraying with Dragendorff’s reagent for an alkaloid detection.

### 3.2. Plant Materials

*Beilschmiedia brevipes* (Lauraceae), collected from Lenggor, Kluang, Johor, Malaysia was identified by Mr Teo Leong Eng. A voucher specimen (KL4978) is deposited at the Herbarium of the Department of Chemistry, University of Malaya, Kuala Lumpur, Malaysia and at the Herbarium of the Forest Research Institute, Kepong, Malaysia. 

### 3.3. Extraction and Isolation of the Alkaloids

The dried leaves (5.0 kg) of *Beilschmiedia brevipes* were ground and extracted exhaustively with hexane (25.0 *l*) for 19 hours. The residual plant material was dried and left for 2 h after moistening with 25% NH_4_OH. It was then macerated with CH_2_Cl_2_ (25.0 *l*) twice for 3-days. After filtration, the supernatant was concentrated to 500 mL at room temperature (30 °C) followed by acidic extraction with 5% HCl until a negative Mayer’s test result was obtained. The aqueous solution was made alkaline to pH 11 with NH_4_OH and re-extracted with CH_2_Cl_2_. This was followed by washing with distilled H_2_O, dried over anhydrous sodium sulphate, and evaporation to give an alkaloid fraction (55.19 g). The crude alkaloid (50.0 g) was submitted to exhaustive column chromatography over silica gel (column dimension = 5 cm, length = 1 m, silica gel 60, 70–230 mesh ASTM; Merck 7734) using CH_2_Cl_2_ gradually enriched with methanol (1% until 20% MeOH; volumes of eluent; 500.0 mL were used for each percentage) to yield 121 fractions. Fractions were then recombined on the basis of their TLC behavior to obtain eight fractions. Fractions 5–6 (7.6 g), afforded an alkaloid identified as (6,7-dimethoxy-4-methylisoquinolinyl)-(4’-methoxyphenyl)-methanone (**1**) (1.8%, PTLC Merck KGaA silica gel 60 F_254_; CH_2_Cl_2_-MeOH; 98:2). Fraction 1 (2.7 g) produced *O*,*O*-dimethylcoclaurine (0.3%) using PTLC (Merck KGaA silica gel 60 F_254_; CH_2_Cl_2_-MeOH; 95:5) [12] and alkaloids **2**-**3** identified as *O*,*O*-dimethylannocherin A (**2**) (0.3%) and (6,7-dimethoxyisoquinolinyl)-(4’-methoxyphenyl)-methanone (**3**) (0.4%), respectively [13]. *O*-Methylvelucryptine (0.3%) [14], armepavine (1.2 %) [15] and norarmepavine (0.2%) [16] were also separated by preparative TLC of fraction 2 (11.6 g) over silica gel using CH_2_Cl_2_-MeOH; 95:5: saturated with NH_4_OH as eluent.

*(6*,*7-Dimethoxy-4-methylisoquinolinyl)-(4’-methoxyphenyl)-methanone* (**1**,**Figure 1**). Brownish amorphous solid; UV max (MeOH): 302 (3.51) nm; IR bands (KBr): 2,929, 1,660, 1,597, 1,521, 1,206, 1,136, 1,031 cm^−1^; ^1^H-NMR (400 MHz, CDCl_3_) and ^13^C-NMR (100 MHz, CDCl_3_): See Table 1; HRESIMS *m*/*z*: 338.1373 [M + H]^+^ (calc. 338.1392 for C_20_H_20_NO_4_).

*O*,*O-Dimethylannocherin A* (**2, Figure 1**), 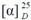 = +91° (*c* = 0.22, MeOH), was obtained as a brownish amorphous solid; UV max (MeOH): 306 (3.46) nm; IR bands (KBr): 3,387, 2,939, 2,833, 1,597, 1,510, 1,430, 1,263, 1,137; ^1^H-NMR (400 MHz, CDCl_3_) and ^13^C-NMR (100 MHz, CDCl_3_): See Table 1; HRESIMS *m*/*z*: 326.1396 [M + H]^+ ^(calc. 326.1392 for C_19_H_20_NO_4_).

### 3.3. Cytotoxic Assays

The cytotoxicity of all the alkaloids was tested against P-388 murine leukemia cells using the MTT-microculture tetrazolim assay [21]. 

## 4. Conclusions

To the knowledge of the authors, alkaloid **1** is the first benzylisoquinoline alkaloid bearing a methyl group at C-4 reported in the family of Lauraceae and this is the first communication on alkaloids from the *Beilschmiedia brevipes*. Alkaloid **2**, which was identified in this study belongs to the rare C-α hydroxybenzylisoquinoline class. Only two examples of this type of alkaloid have been previously reported from *Ocotea pulchella* (Lauraceae) and *Annona cherimola* (Annonaceae), respectively [18,20]. According to Botega *et al.* [20], this type of alkaloids tends to oxidize easily to the corresponding ketones in air therefore alkaloid **3** is most probably formed through oxidation of alkaloid **2**. 
